# Novel Taiwan-I-type infectious bronchitis virus: First evidence of testicular atrophy and efficacious protection with strain-matched oil-emulsified inactivated vaccine

**DOI:** 10.1080/21505594.2026.2664950

**Published:** 2026-04-26

**Authors:** Taoni Zhang, Yangziyu Xie, Chuanrui Yang, Ming Guan, Cheng Lu, Zixue Lin, Jiening Li, Jinlong Dai, Chengyu Zhang, Riwang Yang, Qianhui Wu, Qi Wang, Yi Li, Qin Wu, Tianchao Wei, Jianni Huang, Teng Huang, Meilan Mo

**Affiliations:** a College of Animal Science and Technology, Guangxi University, Nanning, China; b Guangxi Zhuang Autonomous Region Engineering Research Center of Veterinary Biologics, Nanning, China; c Guangxi Key Laboratory of Animal Reproduction, Breeding and Disease Control, Nanning, China

**Keywords:** Infectious bronchitis virus, Taiwan-I-type strain, genome characteristics, testicular atrophy, male reproductive pathology, oil-emulsified inactivated vaccines

## Abstract

The Taiwan-I-type infectious bronchitis virus (IBV) has become one of the most dominant and threatening genotypes circulating in China poultry farms. In this study, we characterized the Taiwan-I-type IBV strain GX-NN200723 and evaluated its pathogenicity and vaccine potential. Phylogenetic and recombination analyses revealed that the strain shared the highest nucleotide similarity with the vaccine strain QXL87 and originated from a recombination event between CK/CH/LSC-99I (major parent) and TW2575/98 (I) (minor parent) within the S1 gene region. Notably, epitope mapping showed significant differences in the number and sequence of antigenic sites compared to commonly used vaccine strains (H120, 4/91, QXL87, LDT3-A), and these differences may largely account for the poor cross-protection and frequent vaccine failures observed in the field. Pathogenicity assessment in 7-day-old SPF chicks demonstrated broad tissue tropism of the strain, causing 30% mortality along with severe reproductive disorders, notably, testicular atrophy in males was observed for the first time in Taiwan-I-type IBV. Furthermore, an oil-emulsified inactivated vaccine (OEIV) developed from the GX-NN200723 strain induced strong humoral and cell-dependent immune responses. Protective efficacy was only evaluated against homologous challenge in this study, the GX-NN200723-OEIV group exhibited 100% survival, the mildest clinical signs, minimal pathological damage, and the lowest viral shedding among all immunized groups. These findings support the use of the GX-NN200723-OEIV as a promising candidate vaccine for controlling Taiwan-I-type IBV infection. This study provides critical insights into the evolution, pathogenesis, and immunization strategies against this economically significant poultry pathogen.

## Introduction

Avian infectious bronchitis (IB) is an acute and highly contagious respiratory disease caused by infectious bronchitis virus (IBV) in chickens, affecting both the respiratory and urogenital systems of chickens with different ages [1], resulting in significant economic losses to the poultry industry. IBV is very prone to mutation, resulting in complex genotypes and serotypes of IBV isolates and poor cross-protection between serotypes of IBVs. Additionally, the significant climatic and environmental variations, chickens with different breeds and ages located in the same areas, diversified farming practices, ineffective biosafety, and live bird trade have collectively contributed to the frequent occurrence of IB on farms, resulting in increasingly complex situations regarding the transmission and prevalence of IBV in production [2]. IBV belongs to the order *Nidovirales*, family *Coronaviridae*, and genus *Coronavirus*, with its genome encoding four structural proteins: the spike (S) protein, envelope (E) protein, membrane (M) protein, and nucleocapsid (N) protein [3]. Among them, the S protein is the primary structural protein of IBV, which is cleaved into S1 and S2 subunits after translation, facilitating the binding of the virus to its receptor and subsequent entry into host cells [4]. The S1 protein contains an independent receptor-binding domain that can induce neutralizing antibodies, inhibit blood clot formation, and generate epitopes for serotype-specific antibodies [4]. Currently, the nucleotide sequence analysis of the S1-encoding region is commonly utilized to determine the genetic type of IBV. Based on the S1 gene, IBV currently circulating worldwide can be classified into at least 9 genotypes and 41 lineages [5]. There are four major lineages coexist in China, including QX-type (GI-19), Taiwan-I-type (GI-7), 793B-type (GI-13), and TC07-2 (GVI-1) [6]. Among them, the Taiwan-I-type strain is prevalent in nearly 20 regions, covering major chicken-raising areas in China, with over 220 isolates [7]. Given its widespread prevalence, whole-genome analysis of current Taiwan-I-type isolate is essential for tracking viral evolution and informing effective control strategies.

IB manifests in multiple forms – respiratory, nephropathic, reproductive, enteric, and proventricular, depending on the tissue tropism of virus [8]. The Taiwan-I-type IBV was first identified in Taiwan, China, in 1996 [9] and was introduced to mainland China around 2009, likely through poultry trade and migratory birds, eventually establishing itself as a dominant genotype [2]. However, studies on its pathogenicity remain limited. The previous reports described Taiwan-I-type strains causing tracheitis, nephritis, urate deposition, cystic oviduct lesions, and reduced egg quality in young hens, leading to severe economic losses [10,11]. Due to the high mutation and recombination rates of IBV [12–14], prevention has become increasingly challenging, underscoring the need for updated and systematic studies on newly emerging Taiwan-I-type strains.

The immunodominant S1 protein drives protective immunity, but its variability often limits cross-protection between heterologous strains [1]. As Taiwan-I-type IBV continues to spread, vaccines tailored to this genotype are urgently needed. Although several live attenuated Taiwan-I-type vaccines have been developed, such as TW2575/98 (P74) [15], CK/CH/GD/GZ14 (P105) [16], and aGD (P140) [17], an oil-emulsified inactivated vaccine (OEIV) targeting this genotype remains lacking. OEIVs offer advantages including improved safety, stable antigen content, and the potential to address antigenic drift. Given their well-established production process, developing a Taiwan-I-type-specific OEIV could significantly enhance IB control.

A Taiwan-I-type IBV strain GX-NN200723 was recently isolated by our group. The genetic characteristics, pathogenic potential, and vaccine applicability have not been fully characterized. In this study, we performed high-throughput whole-genome sequencing of GX-NN200723 and analyzed its nucleotide similarity, recombination patterns, and B-cell epitopes to elucidate its molecular characteristics. We further assessed its pathogenicity in specific-pathogen-free (SPF) chickens and evaluated its suitability as a candidate strain for an OEIV. Our work provides critical insights and a theoretical foundation for the prevention and control of Taiwan-I-type IBV infections.

## Materials and methods

### Virus and virus titration

The Taiwan-I-type strain GX-NN200723 [18] (GenBank accession number: PX488445) was isolated from 55-day-old local breed of chickens with obvious symptoms of acute respiratory disease and nephritis in July 2020. The diseased chickens had previously been vaccinated with live vaccine strain H120 at 1 and 12 d old, as well as with live vaccine strains H120, 4/91 and QXL87 at 30 d old. The isolate was propagated in specific pathogen free (SPF) embryonated chicken eggs and identified as IBV by RT-PCR and sequencing, and subsequently titrated. The viral titer, expressed as the 50% tracheal organ ring culture infection dose (TOC-ID_50_), was determined using the Reed–Muench method [19].

### Animals and ethics statement

SPF embryonated chicken eggs were purchased from Beijing Boehringer Ingelheim Vital Biotechnology Co. Ltd. (Beijing, China). 18–20-day-old embryonated chicken eggs purchased from Guangxi Fufeng Farming and Animal Husbandry Group Co. Ltd. (Guangxi, China). 1-day-old SPF chicks were purchased from Guangxi Li Cheng Dong Biological Co. Ltd. (Guangxi, China). 1-day-old Three-Yellow chickens were purchased from Guangxi Zhushi Agricultural and Livestock Co. Ltd. (Guangxi, China). All the animal rearing conditions are in accordance with the standards. At the experimental endpoint, all animals were humanely euthanized in accordance with the American Veterinary Medical Association (AVMA) Guidelines for the Euthanasia of Animals. Specifically, both chicken embryos and live chickens were euthanized using carbon dioxide (CO_2_) in a manner consistent with AVMA-recommended procedures. The animal study protocol (approval number: GXU‑2024‑293) was reviewed and approved by the Animal Care and Welfare Committee of Guangxi University. The study was reported in accordance with the ARRIVE guidelines 2.0, and the corresponding checklist is provided as Supplementary File.

### Whole genome sequence analysis

The viral RNA was extracted from allantoic liquid by Trizol method, and the cDNA was obtained by reverse transcription using the HiFiScript cDNA Synthesis Kit (ComWin Biotech Co. Ltd., Beijing, China). The complete genome sequence of the GX-NN200723 stain was obtained through high-throughput sequencing by Shanghai Genergy Biotechnology Co., Ltd. (Shanghai, China) using the Illumina HiSeq™ 3000 platform. This platform utilizes Illumina’s sequencing-by-synthesis technology to generate high-quality genome data. The nucleotide similarities between the GX-NN200723 strain and the commonly used commercial live attenuated vaccines (H120, 4/91, QXL87, LDT3-A) were analyzed with MegAlign software. Recombination events were analyzed using RDP4 software (version 4.96) with default settings. Six algorithms, including RDP, GENECONV, BootScan, MaxChi, Chimaera, and 3Seq, were used to detect potential recombination events, with a significance threshold of *p* < 0.05. Results were further confirmed using SimPlot version 3.5.1 with a window size of 200 nucleotides, step size of 20 nucleotides, Kimura 2-parameter model, and 1000 bootstrap replicates. The B-cell epitope was analyzed with IEDB software online. The representative reference strains are shown in Table 1.

**Table 1.** Details of IBV reference strains.

IBV strains	Years of isolation	Country of origin	Accession numbers
M41	Vaccine	USA	GQ504725
H120	Vaccine	Netherlands	GU393335
H52	Vaccine	Netherlands	EU817497
Ma5	Vaccine	USA	KY626045
4/91	Vaccine	UK	KF377577
Arkansas DPI	1981	USA	GQ504720
Beaudette CK	NA	UK	AJ311317
Conn46 1972	1972	USA	FJ904717
Delaware 072	1992	USA	GU393332
Cal99	1999	USA	AY514485
Gray	1960	USA	GU393334
Georgia	1998	USA	GQ504722
Iowa	1956	USA	GU393337
JMK	1964	USA	GU393338
Holte	1954	USA	GU393336
ITA/90254/2005	2005	Western Africa	FN430414
IBV-EP3	NA	Singapore	DQ001338
TW2575/98	1998	China,Taiwan	DQ646405
LX4	1999	China	AY338732
KM91	1991	Korea	JQ977698
SNU8067	2008	Korea	JQ977697
A2	1996	China	EU526388
ZJ971	1997	China	EU714028
BJ	1997	China	AY319651
SAIBK	NA	China	DQ288927
LDT3-A	2003	China	KR608272
CK/CH/LSC/99I	1999	China	KY799582
ck/CH/LDL/97I substrain P115	2001	China	JX195178
ck/CH/LDL/97I substrain P5	1997	China	JX195177
CK/CH/LHLJ/95I	2013	China	KX185057
Peafowl/GD/KQ6/2003	2003	China	AY641576
Partridge/GD/S14/2003	2003	China	AY646283
QXL87	2013	China	PP100176

### Evaluation of pathogenicity test

To evaluate the pathogenicity of the Taiwan-I-type IBV, a total of 200 7-day-old SPF chickens (equal numbers of rooters and hens) were randomly divided into two groups, the Taiwan-I group (*n* = 120) and the control group (*n* = 80). The Taiwan-I group were inoculated with allantoic fluid containing 10^6^ TOC-ID_50_ of the Taiwan-I-type strain GX-NN200723 *via* the ocular-nasal route, while the control group received an equal volume of negative allantoic fluid in the same manner. Clinical signs and survival were monitored for 14 d post-infection (dpi) using a standardized scoring system [20]. For pathological evaluation, 10 chickens per group (sex-balanced) were euthanized at 3, 5, 7, 9, 11, 14, and 133 dpi. Gross lesions were examined in the trachea, kidneys, and reproductive tissues (ovary, oviduct, testis). Tissue samples from these tissues were collected, fixed, and processed for histopathological analysis by hematoxylin and eosin (H&E) staining. Additionally, viral RNA loads in the trachea, kidney, cecal tonsil, bursa, cloacal swabs, and reproductive tissues (ovary, oviduct, testis) collected from 3 to 14 dpi and at 133 dpi were quantified using real-time PCR as previous description [21]. The outcome assessment in this animal study was conducted using a double-blinded method.

### Evaluation of vaccine efficacy

#### Immunization and challenge

The oil-emulsified inactivated vaccine (OEIV) was prepared using the inactivated Taiwan‑I‑type strain GX‑NN200723 and three commercial vaccine strains (H120, 4/91, and QXL87) formulated in a water‑in‑oil emulsion. Briefly, the aqueous phase was prepared by mixing inactivated viral solution with Tween-80 at a ratio of 96:4. The oil phase consisted of Span-80 and Marcol 52 white oil at a ratio of 1:10. The two phases were then combined by gradually adding the aqueous phase into the oil phase at a 2:3 ratio under vigorous shaking to achieve complete emulsification. A total of 120 one‑day‑old healthy Three-Yellow chickens were randomly assigned to six groups (20 chickens per group, equal numbers of rooters and hens). Four groups were immunized subcutaneously with 0.5 mL of the corresponding OEIV (GX‑NN200723‑OEIV, H120‑OEIV, 4/91‑OEIV, and QXL87‑OEIV) at 14 and 28 d of age. The remaining two groups served as unvaccinated‑challenged group and control group. At 42 d of age, all groups except the control group were challenged with 10^6^ TOC-ID_5__0_ of the GX‑NN200723 strain *via* the ocular‑nasal route. A detailed experimental design is provided in Table 2. The outcome assessment in this animal study was conducted using a double-blinded method.

**Table 2.** Experimental design of the immunization and challenge tests against GX-NN200723 strain in chickens.

Groups	No.of chickens	Age (d)	Vaccines	Routes	42 d Challenge	Route and dose of challenge
GX-NN200723-OEIV	20	14 + 28	GX-NN200723-OEIV	Subcutaneous injection in the neck	GX-NN200723	Oculo-nasally, 10^6^ TOC-ID_50_/bird, 0.2 mL
H120-OEIV	20	14 + 28	H120-OEIV		GX-NN200723	
4/91-OEIV	20	14 + 28	4/91-OEIV		GX-NN200723	
QXL87-OEIV	20	14 + 28	QXL87-OEIV		GX-NN200723	
Unvaccinated-challenged	20	14 + 28	–	–	GX-NN200723	
Control	20	–	–	–	–	–

Note: “-” indicates that no operation has been performed.

### Detection of IBV-specific antibodies

Serum samples were collected from five chickens per group at 0, 7, 14, 21, and 28  post‑vaccination (dpv). IBV‑specific serum antibodies were subsequently measured using a commercial ELISA kit (IDEXX, Westbrook, USA) following the manufacturer’s instructions.

### Detection of IBV-neutralizing antibodies

Serum neutralizing antibody titers against IBV were measured in immunized chickens at 0, 14, and 28 dpv using a TOC‑based neutralization assay as previously described [21]. The neutralization titer is determined by the highest dilution of serum that can neutralize the virus and thereby prevent the occurrence of ciliostasis and abnormal ciliary beating.

### Detection of CD3^+^, CD4^+^ and CD8^+^ T lymphocytes by flow cytometry

Five chickens were randomly selected from each experimental group for peripheral blood samples collection *via* the wing vein at 0, 7, 14, 21, and 28 dpv. The preparation of peripheral blood lymphocytes and the analyses of percentages of CD3^+^, CD4^+^ and CD8^+^ T lymphocytes were performed based on established protocols [21] and the manufacturer’s instructions. Data were subsequently processed and summarized using FlowJo 10.8.1 software.

### Detection of cytokine assay

Five blood samples were collected from the wing veins of each group at 0, 14 and 28 dpv. The concentrations of cytokines IL-4 and IFN-γ in the sera were detected according to the instructions for the enzyme immunoassay kit (Meimian Industrial, Jiangsu, China).

### Assessment of clinical signs, gross lesions, and histopathology

Following challenge, clinical signs and survival were monitored daily for 14 d. At 5 d post-challenge (dpc), five chickens per group were euthanized for gross examination of the trachea and kidneys, corresponding tissue samples were collected for histopathological processing. At 98 dpc, five chickens per group were euthanized to examine whether developmental retarline, dation, or gross lesions occurred in the reproductive organs (ovaries, oviducts, testes), and to conduct similar histopathological evaluations on the tissues.

### Assessments of ciliary activity

At 5 dpc, five chickens per group were euthanized, and then the tracheal rings were prepared for ciliostasis test. The protection scores were calculated according to the previous description [22].

### Detection of virus loads

Five tracheas and kidneys were collected from each group at 5 dpc, and five oral swabs and cloacal swabs were collected at 1, 3, 5, 7, 11, and 14 dpc. The virus loads in these samples were detected by real-time PCR as previous description [21].

## Statistical analysis

All data were visualized and plotted using GraphPad Prism version 8.0.1 software. Statistical analyses were performed using IBM SPSS Statistics version 25 software. The Shapiro–Wilk normality test was performed prior to all ANOVA analyses to confirm data normality, ensuring the statistical methods were appropriately applied. For comparisons among multiple groups, one-way analysis of variance (ANOVA) was applied, followed by Duncan’s multiple range test for post-hoc comparisons. For data measured at multiple time points (including viral loads and T cell subsets), repeated-measures ANOVA was used to evaluate the effects of time and group differences. For survival analysis, survival curves were compared using the Log-rank (Mantel-Cox) test. ** indicates the highly significant difference (*p* < 0.01) between each immunized groups and the non-immunized group; * indicates the significant difference (*p* < 0.05) between each immunized group and the non-immunized group. Different letters indicate significant differences (*p* < 0.05) among the experimental groups, while the same letters indicate no significant difference (*p* > 0.05) among them. In addition, in the analysis of animal experiment data, no animals, experimental units, or data points from any group were excluded.

## Results

### Whole genome sequencing and analysis

#### Genomic characterization of the GX-NN200723 strain revealed a classic genome structure

The high-throughput whole-genome sequencing showed that the genome of the GX-NN200723 strain consistent with the genomic structure of the classical strains of IBV, which was 5’UTR-Pol-1a/ab-S-3a-3b-E-M-5a-5b-N-3’UTR. The whole-genome nucleotide sequence length was 27,662 nt (Table 3).

**Table 3.** Gene annotation of complete genome sequence of the GX-NN200723 strain.

Gene	Genome position	Size (nucleotide)	Size (amino acid)
5’−UTR	1–506	506	–
1ab polyprotein	507–20,401	19,895	6632
Spike glycoprotein	20,352–23,846	3,495	1165
3a protein	23,846–24,019	174	58
3b protein	24,019–24,210	192	64
Envelope protein	24,194–24,520	327	109
Membrance protein	24,492–25,169	678	226
5a protein	25,533–25,730	198	66
5b protein	25,727–25,975	249	83
Nucleocapsid protein	25,918–27,147	1,230	410
3’−UTR	27,148–27,662	515	–

Note: “-” indicates not applicable (non-coding region).

#### Amino acid deletions at positions 19 and 23 in S protein of the GX-NN200723 strain

**Figure 1.**
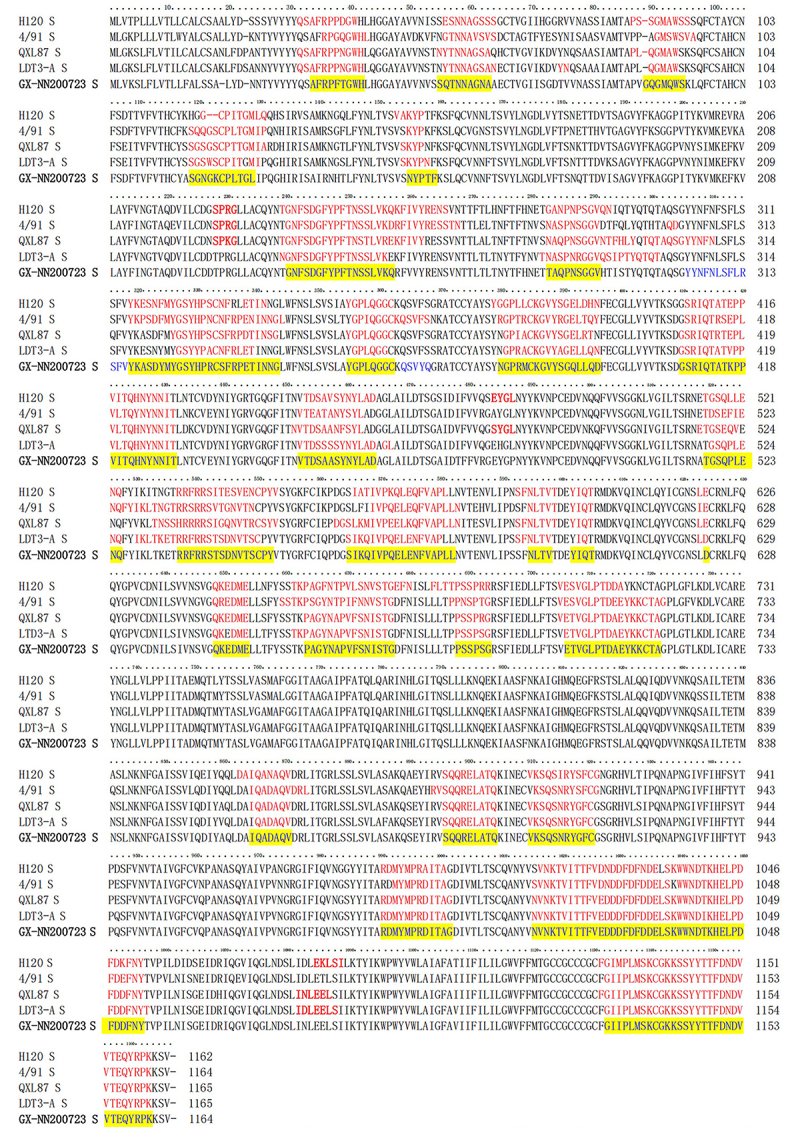
Prediction of B-cell antigen epitopes in the GX-NN200723 strain and commonly administered domestic vaccine strains.

Note: Red indicate vaccine strain epitopes, blue indicate GX-NN200723 epitopes, yellow shading indicate conserved epitopes.

The nucleotide similarities between the GX-NN200723 strain and the commercial vaccine strains ranged from 86.1% to 93.8% for the whole genome, 81.9% to 92.5% for the S gene, 77.4% to 83.9% for the S1 gene, and 85.8% to 99.4% for the S2 gene (Table 4). At the whole-genome level, the GX-NN200723 strain shared the highest nucleotide similarity with QXL87 strain, while at the S gene level, it exhibited the closest identity to LDT3-A strain. The deduced amino acid sequence of the S gene revealed that, the GX-NN200723 strain contained deletions at positions 19 and 23 in the S protein compared to the commercial vaccine strain (Figure 1).

**Table 4.** Nucleotide similarities between the GX-NN200723 strain and commonly used commercial live attenuated vaccines.

strains	whole genome	S gene	S1 gene	S2 gene
H120	86.3%	84.2%	81.7%	86.5%
4/91	86.1%	81.9%	77.4%	85.8%
QXL87	93.8%	86.7%	78.3%	94.4%
LDT3-A	90.7%	92.5%	83.9%	99.4%

#### S1 gene recombination defines the origin of the GX-NN200723 strain

**Figure 2.**
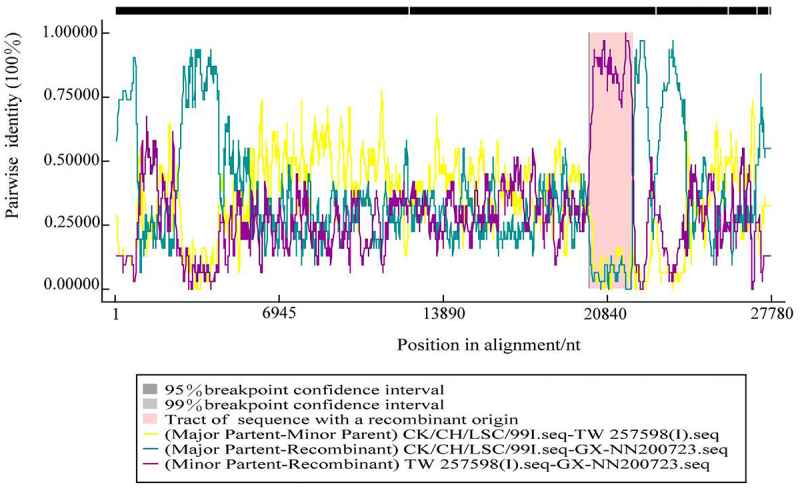
The recombination result of the GX-NN200723 strain was analyzed by RDP4 software.

**Figure 3.**
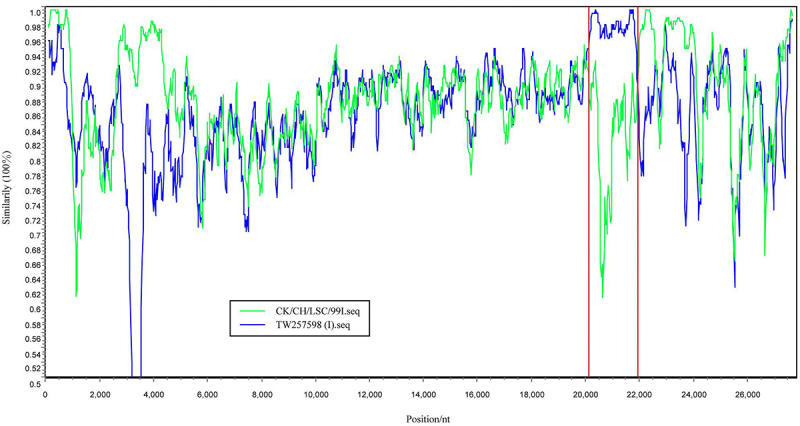
The recombination result of the GX-NN200723 strain was analyzed by SimPlot software.

Recombination event of the GX-NN200723 strain was detected by RDP 4.96 and SimPlot 3.5.1 softwares. The results indicated that the GX-NN200723 strain originated from the recombination between the reference strains CK-CH-LSC-99I (major parent) and TW257598 (I) (minor parent) (Figure 2 and 3). The recombination region was located within the S1 gene region at nucleotides 20,120 to 21,932. The *p*-values calculated by RDP 4.96 for RDP, GENECONV, BootScan, MaxChi, Chimaera, and 3Seq reached 9.142 × 10^−83^, 3.307 × 10^−83^, 1.850 × 10^−88^, 1.291 × 10^−28^, 6.460 × 10^−32^, and 2.220 × 10^−16^, respectively.

#### The GX‑NN200723 strain carries fewer S protein B‑cell epitopes and lacks vaccine‑specific motifs

The B-cell epitope analysis revealed that the GX‑NN200723 strain carries 29 epitopes within the S protein, in contrast to 33, 30, 31, and 33 epitopes identified in the vaccine strains H120, 4/91, QXL87, and LDT3‑A, respectively (Figure 1). Beyond this numerical discrepancy, comparative alignment showed notable variations in epitope sequence similarities between the GX‑NN200723 and vaccine strains. While the majority of epitopes in GX‑NN200723 strain were conserved with those in the vaccine strains, several vaccine-specific epitope motifs – including “SPRG,” “EYGL,” “SYGL,” and “EKLSI” – were absent in GX-NN200723.

## Evaluation of pathogenicity test

### Multi-organ injury, severely compromising the reproductive system were showed in GX-NN200723 challenged chickens

**Figure 4.**
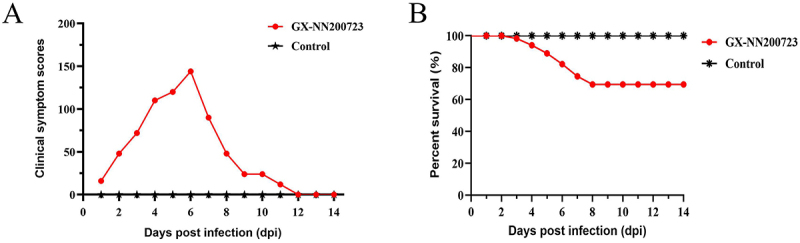
Clinical symptom scores (a) and percentage survival (b) of the SPF chickens challenged with the GX-NN200723 strain.

**Figure 5.**
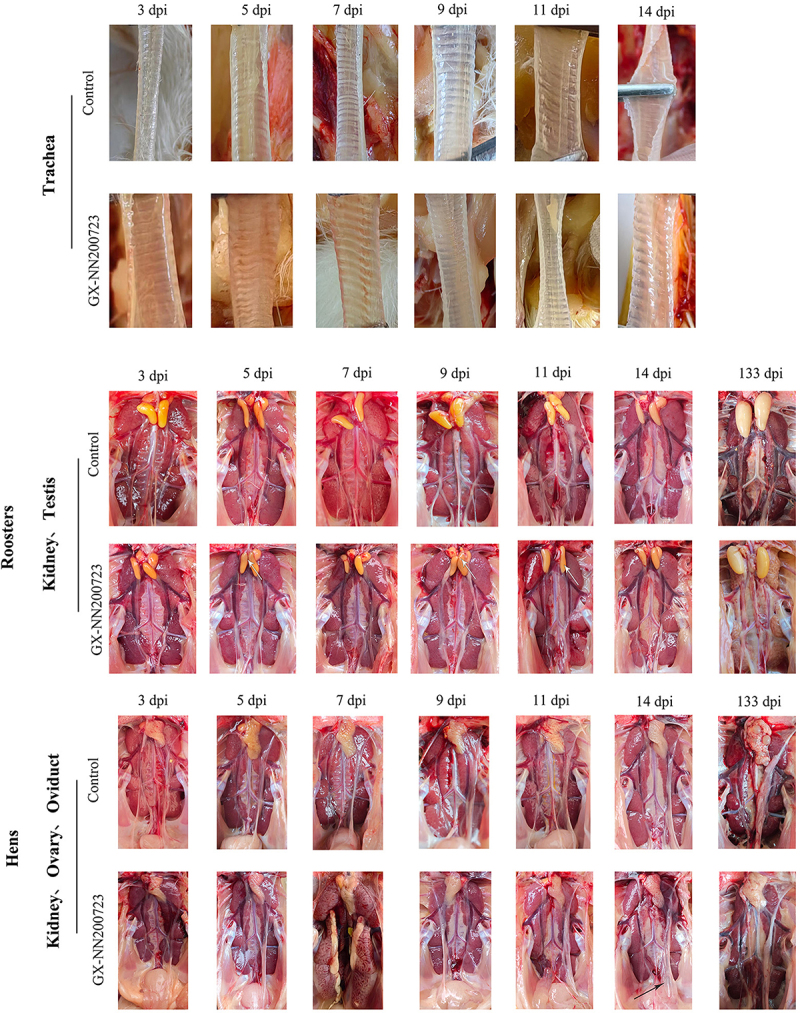
Clinical symptoms of the trachea, kidney, testis, ovary, and oviduct in the GX-NN200723 group and the control group.

Note: white arrows indicate testicular abnormalities or atrophy, black arrows indicate cystic edema.

Following challenge with the Taiwan‑I‑type IBV strain GX-NN200723, the infected SPF chickens exhibited clinical signs of depression, ruffled feathers, tracheal rales, and dyspnea from 1 to 12 dpi, with peak severity at 6 dpi, culminating in a cumulative mortality rate of 30% (36/120) (Figure 4). Gross lesions in these birds included tracheal hemorrhage (5–7 dpi), renal urate deposition (7 dpi), testicular atrophy (5–11 dpi), ovarian hypoplasia (133 dpi), and cystic edema of the oviduct (14 dpi). No clinical signs or gross lesions were observed in the control group throughout the study (Figure 5).

**Figure 6.**
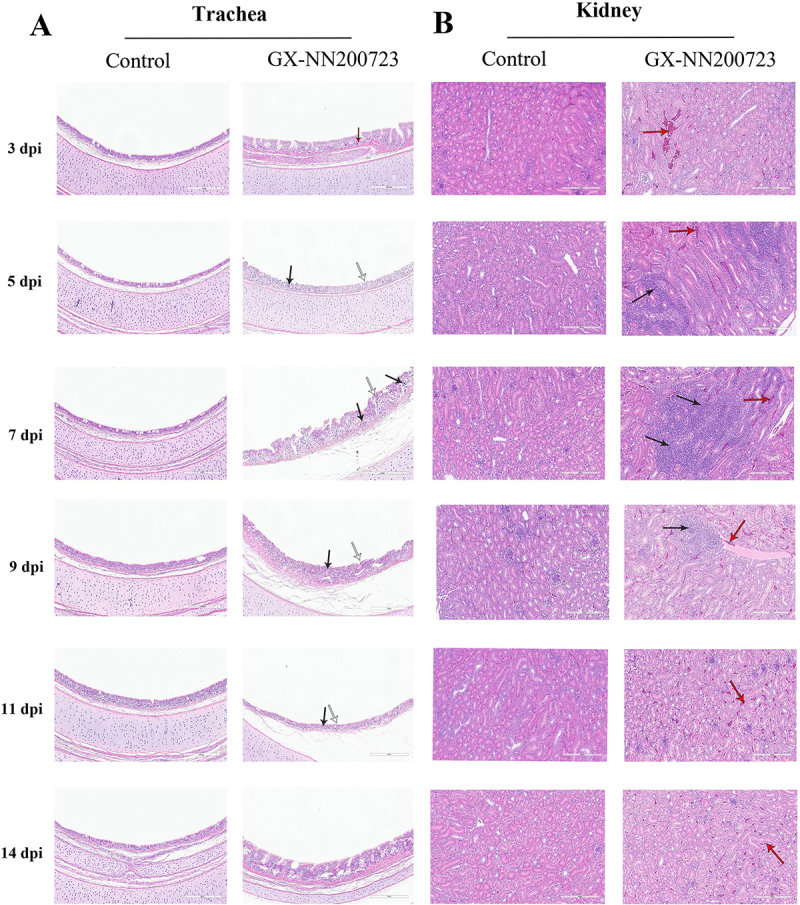
Histopathological analysis of the trachea (a) and kidney (b) in the GX-NN200723 group and the control group.

Note: Black arrows signify lymphocyte infiltration, white arrows depict tracheal cilium shedding, and red arrows denote hemorrhage. Scale bar = 200 μm.

**Figure 7.**
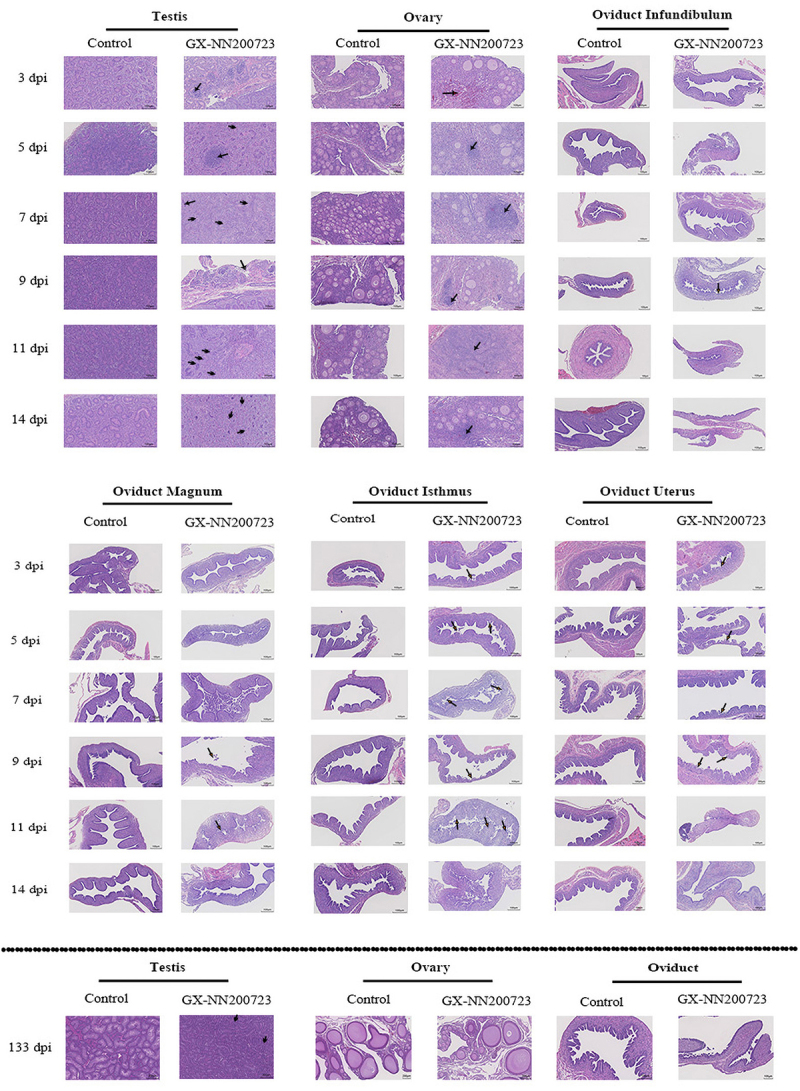
Histopathological analysis of the testis, ovary, and oviduct in the GX-NN200723 group and the control group.

Note: Black arrows indicate lymphocyte infiltration, blue arrows indicate seminiferous tubule atrophy, orange arrows indicate exfoliation of oviduct ciliated epithelial cells, red arrows indicate hemorrhage, and white arrow indicates disorganization of the tunica. Scale bars: 100 μm (testis, ovary, oviduct); 200 μm (testis, ovary) at 133 dpi.

Histopathological analysis revealed severe lesions in the trachea, kidneys, testis, ovary, and oviduct of chickens infected with the GX-NN200723 strain (Figure 6 and 7), while no significant alterations were observed in the control group. Infected tracheas exhibited ciliary loss and extensive inflammatory infiltration persisting until 11 dpi. Renal tissues showed marked tubular dilation, hemorrhage, and interstitial inflammation, most pronounced between 5 and 9 dpi. In the testis, seminiferous tubule atrophy was evident, progressing to persistent atrophy by 133 dpi. There is inflammatory cell infiltration in the ovaries (5 dpi-14 dpi). The oviduct displayed segment‑specific epithelial damage, primarily characterized by ciliary shedding and epithelial exfoliation from 3 to 11 dpi, with no notable pathology remaining at 133 dpi.

### Widespread and prolonged tissue tropism of the GX-NN200723 strain in infected chickens

**Figure 8.**
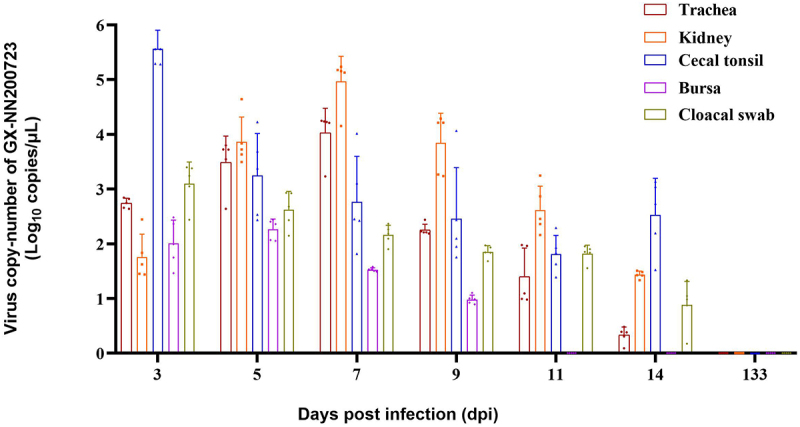
Virus copy-number of the trachea, kidney, cecal tonsil, bursa, cloacal swab in the GX-NN200723 group by real-time PCR.

Note: Sample quantity *n* = 5.

**Figure 9.**
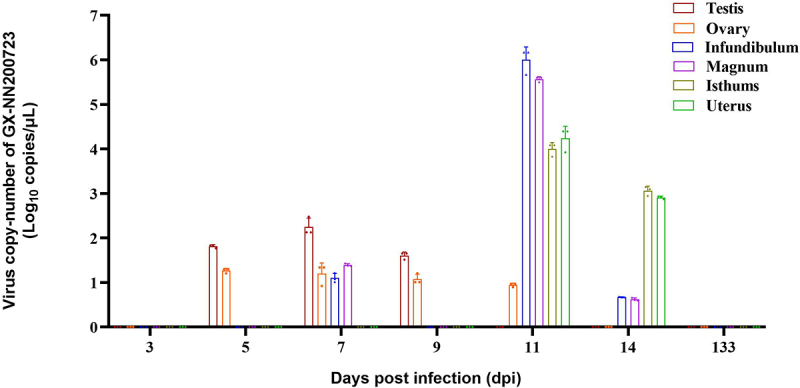
Virus copy-number of the testis, ovary, infundibulum, magnum, isthums, and uterus in the GX-NN200723 group by real-time PCR.

Note: Sample quantity *n* = 3, because 2 out of 5 samples were used for histopathological analysis, and the remaining 3 samples were subjected to RT-qPCR.

Viral RNA loads were detected across multiple tissues in infected chickens (Figure 8 and 9). In the trachea and kidney, the content of viral RNA can be continuously detected at the viral load level within 14 dpi, and reaches its peak in 7 dpi. While the cecal tonsil showed the highest load at 3 dpi, and remained positive throughout 14 dpi period. The bursa of Fabricius was positive from 3 to 9 dpi, and cloacal swabs remained positive until 14 dpi. Within the reproductive tract, viral RNA was present in the testis (5–9 dpi, peak at 7 dpi) and ovary (3–11 dpi). All segments of the oviduct were positive by 11 dpi, with sustained detection through 14 dpi and segment‑specific load differences noted. No viral RNA was detected in any tissue by 133 dpi, and all control samples remained negative throughout the study.

## Evaluation of vaccine efficacy

### IBV-specific antibody titers

**Figure 10.**
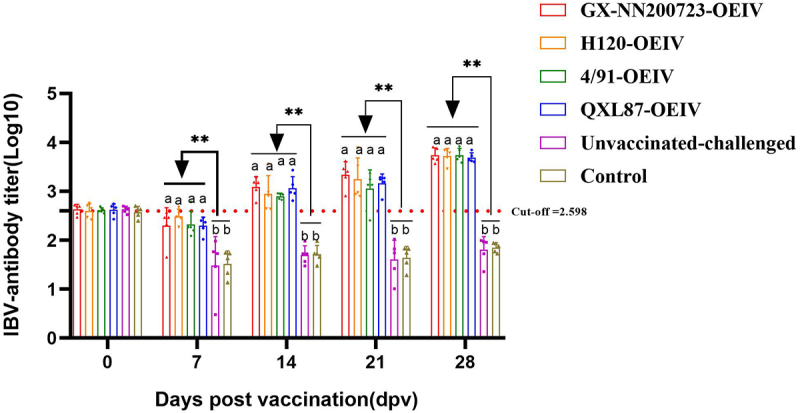
Antibody titers measured in the sera of immunized chickens.

Note: Sample quantity *n* = 5, Cut-off IBV-antibody titer (Log_10_)=2.598, ** indicates the highly significant difference (*p* < 0.01) between each immunized groups and the non-immunized groups. Different letters indicates significant differences (*p* < 0.05) among the experimental groups, while the same letters indicate no significant difference (*p* > 0.05) among them.

IBV‑specific antibody levels in all immunized groups increased progressively from 7 to 28 dpv (Figure 10). Antibody titers were significantly higher in the immunized groups compared to the unvaccinated‑challenged and control groups (*p* < 0.01). However, the antibody response induced by the GX‑NN200723‑OEIV vaccine showed no significant difference from those elicited by the other vaccine formulations (*p* > 0.05).

### IBV-neutralizing antibody titers

**Figure 11.**
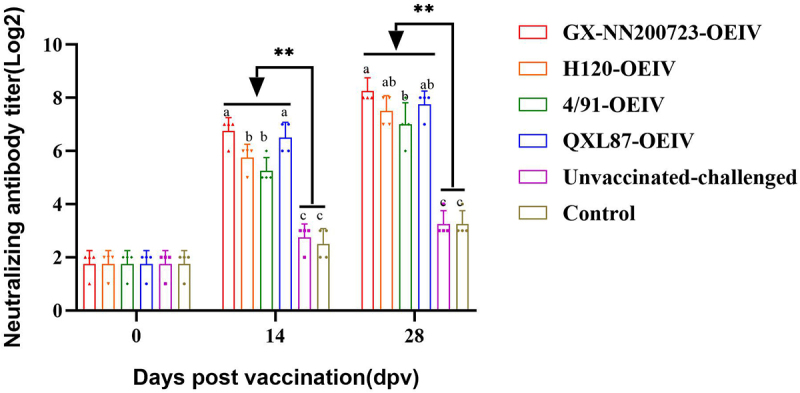
IBV-neutralizing antibody titers measured in the sera of immunized chickens.

Note: Sample quantity *n* = 4. ** indicates the highly significant difference (*p* < 0.01) between each immunized groups and the non-immunized groups. Different letters indicates significant differences (*p* < 0.05) among the experimental groups, while the same letters indicate no significant difference (*p* > 0.05) among them.

The results (Figure 11) showed that neutralizing antibody titers increased progressively over time in all immunized groups, reaching peak levels at 28 dpv, which were significantly higher than those in the unvaccinated‑challenged and control groups (*p* < 0.05). The GX-NN200723‑OEIV group exhibited the highest neutralizing antibody titers at both 14 and 28 dpv. At 14 dpv, its titer was significantly higher than those of the H120‑OEIV and 4/91‑OEIV groups (*p* < 0.05). At 28 dpv, it remained significantly higher than that of the 4/91‑OEIV group (*p* < 0.05).

### Dynamic changes of CD3^+^, CD4^+^, and CD8^+^ T lymphocytes in immunized chickens

**Figure 12.**
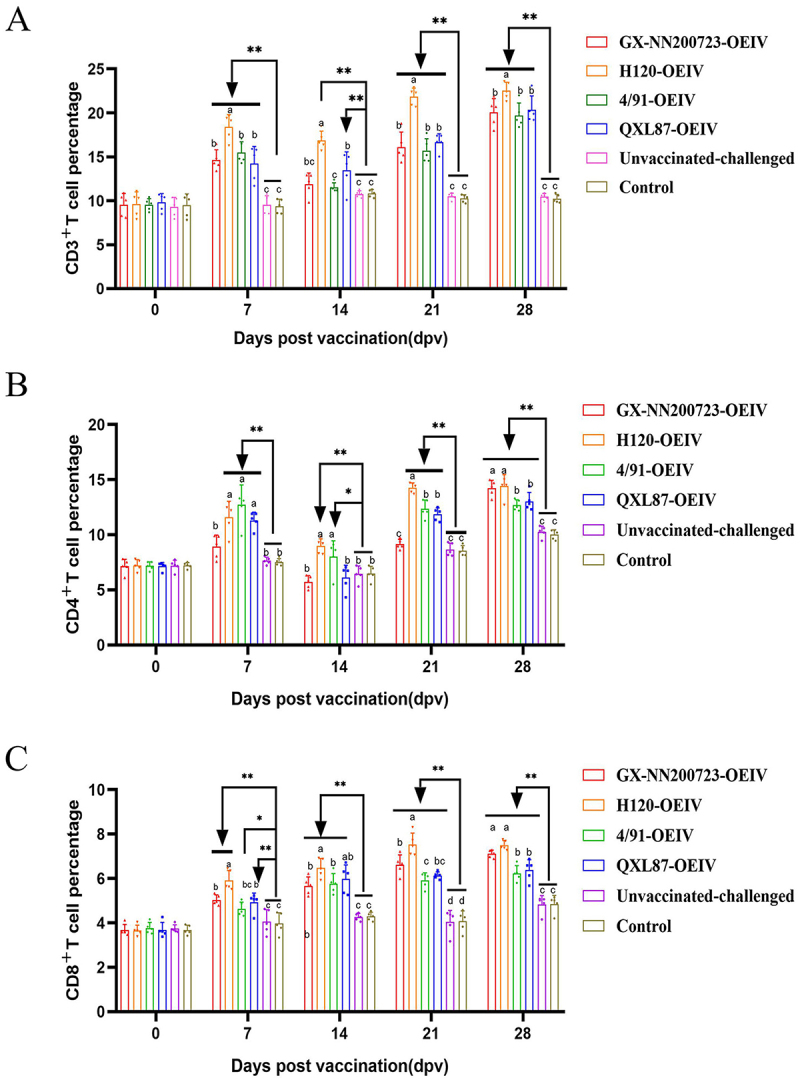
Percentages of CD3^+^ (a), CD4^+^ (b) and CD8^+^ (C) T lymphocytes in peripheral blood of immunized chickens.

Note: Sample quantity *n* = 5, ** indicates the highly significant difference (*p* < 0.01) between each immunized groups and the non-immunized groups; * indicates the significant difference (*p* < 0.05) between each immunized groups and the non-immunized groups. Different letters indicate significant differences (*p* < 0.05) among the experimental groups, while the same letters indicate no significant difference (*p* > 0.05) among them.

The results (Figure 12) indicated that the CD3^+^, CD4^+^, and CD8^+^ T lymphocyte contents in all immunized groups were significantly higher than those in the non-immunized group at different immune time points (*p* < 0.01). The contents of CD3^+^ and CD4^+^ T lymphocytes in all immunized groups began to increase slowly from 7 dpv, decreased slightly at 14 dpv, then increased steadily, and reached the highest level at 28 dpv. The contents of CD3^+^ T lymphocytes in the GX-NN200723-OEIV group showed no significant difference compared to 4/91-OEIV group and QXL87-OEIV group (*p* > 0.05), but were significantly lower than those in the H120-OEIV group (*p* < 0.05). The CD4^+^ T lymphocyte contents in the GX-NN200723-OEIV group were significantly higher than those in the 4/91-OEIV and QXL87-OEIV groups (*p* < 0.05), with no significant difference compared to the H120-OEIV group (*p* > 0.05). The CD8^+^ T lymphocyte contents in all immunized groups gradually increased with the extension of immune time. At 28 dpv, the CD8^+^ T lymphocyte content in the GX-NN200723-OEIV group showed significantly higher than those in the 4/91-OEIV and QXL87-OEIV groups (*p* < 0.05), but there was no significant difference compared with the H120-OEIV group (*p* > 0.05).

### Dynamic changes of IL-4 and IFN-γ in immunized chickens

**Figure 13.**
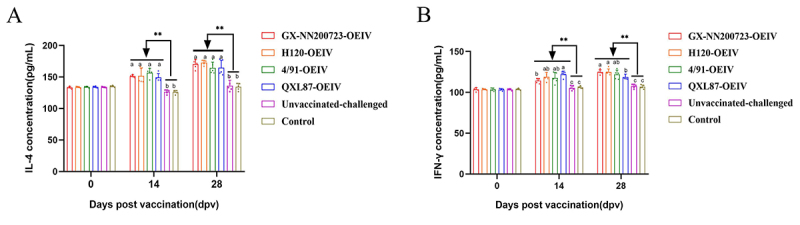
IL-4 (a) and IFN-γ (b) concentrations in the sera of immunized chickens.

Note: Sample quantity *n* = 5, ** indicates the highly significant difference (*p* < 0.01) between each immunized groups and the non-immunized group. Different letters indicate significant differences (*p* < 0.05) among the experimental groups, while the same letters indicate no significant difference (*p* > 0.05) among them.

At 0, 14, and 28 dpv, the concentrations of cytokines IL-4 and IFN-γwere determined. The results (Figure 13) indicated that with the increase in immunization time, the concentrations of IL-4 and IFN-γ in all immunized groups gradually increased and were significantly higher than those in the non-immunized group (*p* < 0.01). At 28 dpv, IL‑4 levels in the GX‑NN200723‑OEIV group were comparable to those in other immunized groups (*p* > 0.05). The IFN-γ content in the GX-NN200723-OEIV group was not significantly different from those in the H120-OEIV and 4/91-OEIV groups (*p* > 0.05), but was significantly higher than that in the QXL87-OEIV group (*p* < 0.05).

### The lowest clinical symptom score and the highest survival rate of 100% were showed in the GX-NN200723-OEIV group

**Figure 14.**
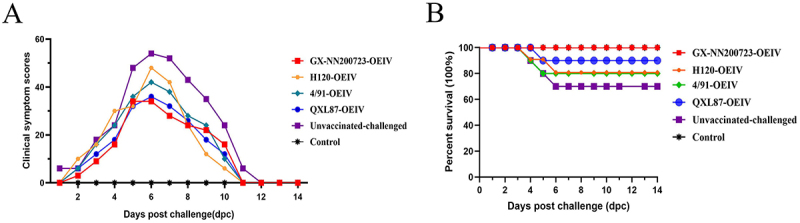
Clinical symptom scores (a) and percentage survival (b) of the Three-Yellow chickens in all experimental groups challenged with the GX-NN200723 strain.

Note: Survival curves were compared using the Log-rank (Mantel-Cox) test.

Clinical symptoms and survival rates of vaccinated-challenged chickens were observed and recorded within 14 dpc. The results (Figure 14) indicated that the clinical symptom scores of the other groups gradually increased and then gradually decreased, except for the control group. Among them, the unvaccinated-challenged group had the longest duration of clinical symptoms, lasting for 12 d, with the highest clinical symptom score and the lowest survival rate of 70%. The duration of clinical symptoms in all immunized groups was 11 d. Furthermore, the GX-NN200723-OEIV group demonstrated a lower overall clinical symptom score compared to the remaining immunized groups, along with the highest survival rate of 100%, which was significantly greater than those observed in other immunization groups.

### No gross lesions, the largest testes, and the longest oviducts were showed in the GX-NN200723-OEIV group

**Figure 15.**
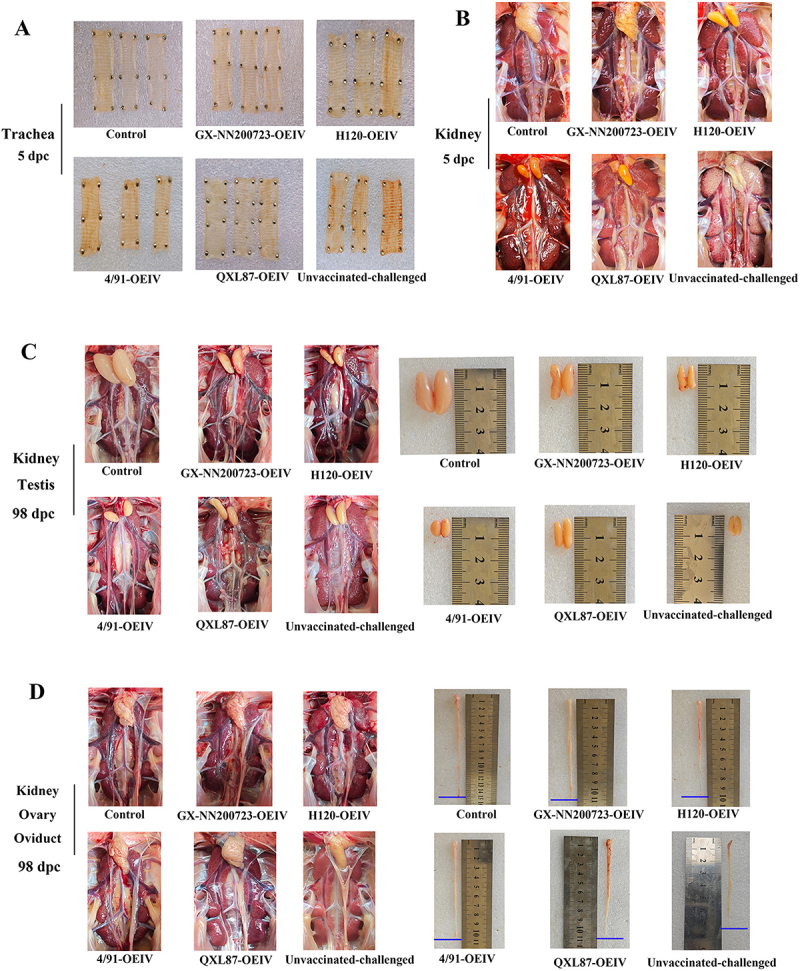
Clinical symptoms of the trachea and kidney in Three-Yellow chickens from all experimental groups challenged with the GX-NN200723 strain.

At 5 dpc, gross lesions were absent in the control group but present in all other groups (Figure 15). The unvaccinated‑challenged group showed the most severe pathology, including marked tracheal hemorrhage and renal urate deposition, while the immunized groups exhibited milder changes. The GX‑NN200723‑OEIV group displaying nearly no tracheal hemorrhage and no significant renal pathology. At 98 dpc, the testicles of all the vaccinated groups were larger than those of the unvaccinated groups), and the oviduct lengths in all the vaccinated groups were longer than those in the unvaccinated groups. The control group showed the largest testes and the longest oviducts among all groups. Among the vaccinated groups, testicular size decreased in the order: GX‑NN200723‑OEIV >QXL87‑OEIV >H120‑OEIV >4/91‑OEIV. For oviduct length, the GX‑NN200723‑OEIV, 4/91‑OEIV, and QXL87‑OEIV groups were comparable, while the H120‑OEIV group was relatively shorter. No obvious macroscopic abnormalities were observed in the ovaries across all groups.

### No histopathological changes in the GX-NN200723-OEIV group

**Figure 16.**
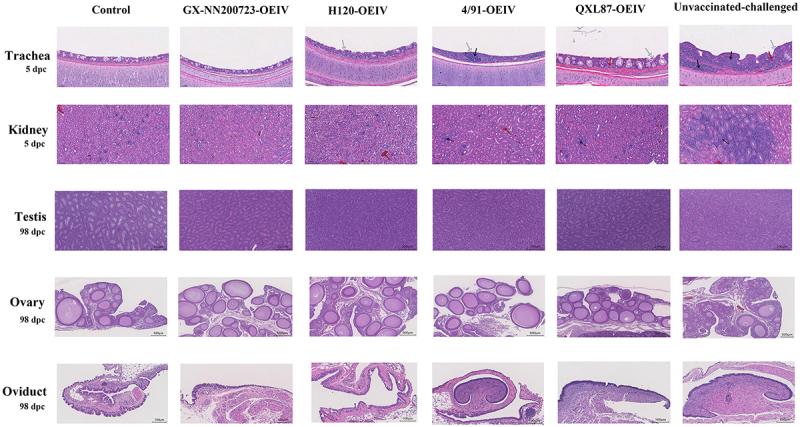
Histopathological analysis of the trachea and kidney in Three-Yellow chickens from all experimental groups challenged with the GX-NN200723 strain.

Note: Black arrows signify lymphocyte infiltration, white arrows depict tracheal cilium shedding, and red arrows denote hemorrhage. Scale bars: 50 μm (trachea, kidney); 200 μm (testis); 500 μm (ovary); 100 μm (oviduct).

Histopathological examination revealed no lesions in the control group, whereas the unvaccinated‑challenged group displayed the most severe pathology, including tracheal ciliary loss, inflammation, and hemorrhage, as well as renal tubular dilation and inflammatory infiltration. The immunized groups showed milder changes: the H120‑OEIV, 4/91‑OEIV, and QXL87‑OEIV groups exhibited tracheal ciliary loss and mild renal hemorrhage or inflammation, while the GX‑NN200723‑OEIV group presented nearly normal tracheal and renal morphology (Figure 16). At 98 dpc, examination of the testis, ovary, and oviduct indicated no significant histopathological alterations in any group compared with the control group (Figure 16).

### Ciliary activity

**Figure 17.**
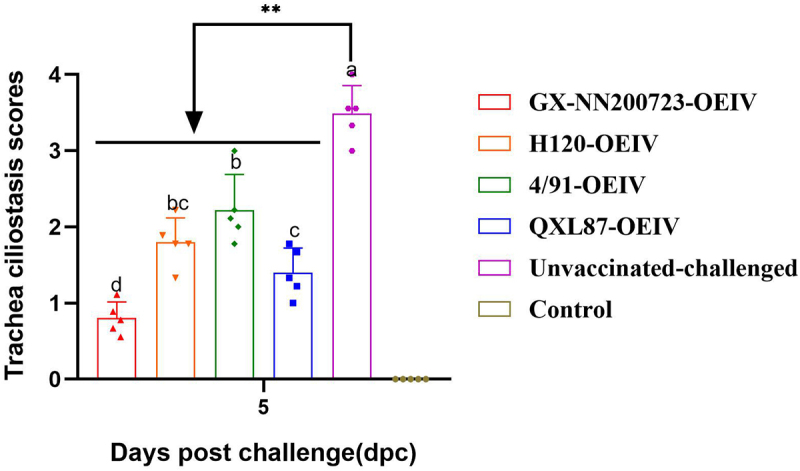
Trachea ciliostasis scores in Three-Yellow chickens from all experimental groups challenged with the GX-NN200723 strain.

Note: Sample quantity *n* = 5, ** indicates the highly significant difference (*p* < 0.01) between Unvaccinated-challenged group and the immunized group. Different letters indicate significant differences (*p* < 0.05) among the experimental groups, while the same letters indicate no significant difference (*p* > 0.05) among them.

At 5 dpc, tracheal ciliary stasis was evaluated and scored in all groups. As shown in Figure 17, the control group exhibited no ciliary impairment, with a ciliary stasis score of zero. In contrast, the unvaccinated‑challenged group displayed the most severe ciliary damage, with a score significantly higher than those of all immunized groups (*p* < 0.01). Among the immunized groups, the protective effect on tracheal cilia was ranked as follows: GX-NN200723-OEIV group, QXL87-OEIV group, H120-OEIV group, 4/91-OEIV group.

### Viral RNA detection

**Figure 18.**
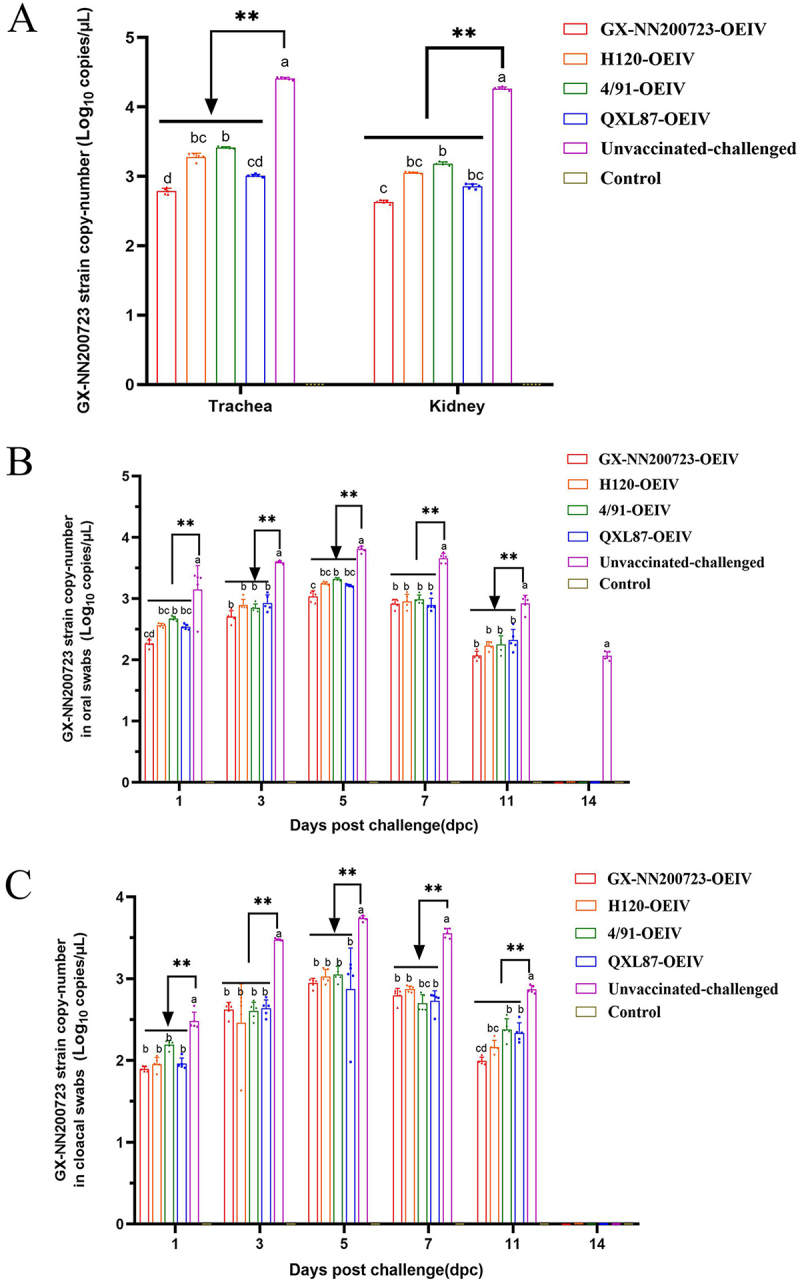
Virus copy-number of the trachea (a), kidney (a), oral swabs (b) and cloacal swabs (C) from all experimental groups challenged with the GX-NN200723 strain.

Note: Sample quantity *n* = 5, ** indicates the highly significant difference (*p* < 0.01) between Unvaccinated-challenged group and the immunized group. Different letters indicate significant differences (*p* < 0.05) among the experimental groups, while the same letters indicate no significant difference (*p* > 0.05) among them.

At 5 dpc, tracheas and kidneys were collected for virus loads detection. The results (Figure 18 A) showed that, except for the control group, in which no virus was detected, the unvaccinated‑challenged group had the highest viral load, which was significantly higher than those of all immunized groups (*p* < 0.05). The GX-NN200723-OEIV group exhibited the lowest viral load, being significantly lower than the 4/91-OEIV group (*p* < 0.05), while showing no statistically significant differences compared to H120-OEIV and QXL87-OEIV groups (*p* > 0.05). Viral loads in oral and cloacal swabs were measured at 1, 3, 5, 7, 11, and 14 dpc. As shown in Figure 18 B and 18 C, viral shedding levels in both oral and cloacal swabs from all groups initially increased and then declined over time. Among the immunized groups, the GX‑NN200723‑OEIV group exhibited the lowest viral loads, although the difference was not statistically significant compared to the other vaccine groups (*p* > 0.05). By 14 dpc, viral shedding was no longer detectable in all immunized groups but persisted in the unvaccinated-challenged group.

## Discussion

IB is one of the main causes of economic losses in the global poultry industry [23]. IBV infection affects the respiratory tract, kidneys, and reproductive system, leading to decreased growth performance, reduced egg production and quality, as well as increased susceptibility to secondary pathogens [1]. The increasing prevalence of Taiwan-I-type IBV has prompted studies on its pathogenicity [10,11] and immunogenicity [15,16]. However, as the characteristics of historical isolates may not fully represent those of currently circulating strains, continuous surveillance of emerging variants – coupled with comprehensive analyses of their genomic features, pathogenicity, and immunogenicity – remains essential.

In the present study, we comprehensively analyzed the complete genome of the Taiwan-I-type IBV strain GX-NN200723, including its genomic structure, nucleotide similarity, recombination events, and B-cell epitope profiles. It is showed that the GX-NN200723 strain retained the classical IBV genomic organization. Whole-genome nucleotide similarity was highest with vaccine strain QXL87, while its S gene was most closely related to LDT3-A. The deduced S protein contained deletions at positions 19 and 23 compared to commercial vaccine strains. Recombination analysis indicated that GX-NN200723 likely originated from recombination between CK/CH/LSC-99I (major parent) and TW257598(I) (minor parent), with a breakpoint located within the S1 gene – a region critical for viral tropism and antigenicity. Such recombination may lead to altered biological properties, and is consistent with the previous report [24]. B-cell epitope prediction revealed marked differences in antigenic sites between this strain and commonly used vaccine strains (H120, 4/91, QXL87, LDT3-A). These findings suggest limited antigenic relatedness, which may explain the frequent vaccine failures and inadequate cross-protection observed in the field. Therefore, developing vaccines tailored to currently circulating Taiwan-I-type strains is essential for improving IBV control strategies.

To investigate the pathogenic characteristics of the Taiwan-I-type IBV strain GX-NN200723, 7-day-old SPF chicks were infected and monitored until 140 d of age. It is showed that the strain caused 30% mortality, consistent with some reports [25,26] but lower than the 80% reported elsewhere [10], a discrepancy possibly attributable to differences in genetic background and recombinant origin. Notably, this is the first report of testicular atrophy in roosters caused by a Taiwan-I-type IBV: infected roosters exhibited seminiferous tubule atrophy by 14 dpi and visibly smaller testes at 133 dpi, resembling lesions induced by QX and Mass genotypes [27–29]. Viral shedding via semen from infected roosters may indirectly affect hens through mating, potentially causing serious losses [30]. In hens, inflammatory cell infiltration in the ovaries and epithelial exfoliation in all oviduct segments (most severely in the isthmus) were observed within 14 dpi, accompanied by cystic edema; by 133 dpi, infected hens showed fewer ovarian follicles and smaller oviducts, indicating severe reproductive impairment. Although various globally prevalent strains (GI-19 [31–33], GI-1 [34,35], GVI-1 [36], and GI-7 [10,11]) cause cystic edema and false layer syndrome, pathogenic profiles can differ even within the same genotype [34,37], influenced by factors, such as strain, age at infection [10], and antibody levels [38]. Viral load dynamics revealed that replication in trachea and kidneys peaked at 7 dpi and remained detectable through 14 dpi. Among lymphoid tissues, cecal tonsils exhibited the highest viral loads from 3 dpi and remained positive throughout the 14-day monitoring period, though no virus was detected at 133 dpi – previous reports indicating Taiwan-I-type IBV can persist in cecal tonsils for up to 200 d^[11]^ suggest this site may serve as a long-term reservoir. The bursa of Fabricius tested positive from 3 to 9 dpi (peak at 5 dpi), consistent with previous findings [39]. Viral shedding via cloacal swabs persisted through 14 dpi and cleared by 21 dpi; however, renewed shedding at 156 dpi has been reported [11,40], implicating the trachea, kidneys, cecal tonsils, and cloaca as potential long-term reservoirs. Notably, this study provides the first continuous detection of viral loads in the reproductive system within 14 d after IBV infection: viral RNA was detected in testes (5–9 dpi, peak at 7 dpi), ovaries (3–11 dpi), and all oviduct segments by 11 dpi, with higher loads persisting in the isthmus and uterus at 14 dpi – virological findings that closely correlated with the observed histopathological lesions, confirming the reproductive tropism of GX-NN200723. This work lays a foundation for further research into the reproductive tropism of Taiwan-I-type IBV strains. A notable limitation of the present study is that viral load detection was not performed at intermediate time points between 14 and 133 dpi. This lack of intermediate sampling creates a gap in delineating the precise dynamics of viral persistence, tissue latency, and clearance during the prolonged post-infection period. Future studies will include additional intermediate sampling time points to systematically track viral loads in tissues, such as the cecal tonsils, reproductive tract, and other potential reservoirs, thereby clarifying the long-term persistence and clearance patterns of Taiwan-I-type IBV.

Just as mentioned above, the absence of viral load data between 14 dpi and 133 dpi represents a key methodological limitation of this study, particularly given our focus on long-term reproductive outcomes. However, to our knowledge, this is the first study to systematically and quantitatively characterize the detectable presence and tissue distribution of IBV in distinct regions of the reproductive system, including the testes, ovaries, and multiple oviduct segments. Further, we found that the viral RNA levels in reproductive tissues were generally lower than those in the trachea, kidneys, cecal tonsils, and bursa of Fabricius within the first 14 dpi. Notably, severe and persistent pathological injury was observed despite viral clearance, rather than resulting from ongoing viral replication. This strongly suggests an indirect rather than direct cytopathic mechanism underlying IBV-induced reproductive system damage. We propose that endocrine disruption may be the key mechanism: Reproductive hormones such as testosterone, luteinizing hormone, and follicle-stimulating hormone play critical roles in maintaining testicular development and spermatogenesis. This hypothesis regarding endocrine disorder remains to be experimentally validated. Future studies focusing on quantitative detection of these hormone levels will help to further verify the endocrine mechanism of IBV-induced testicular atrophy.

Based on our pathogenicity studies, we developed an OEIV targeting the prevalent Taiwan-I-type strain GX-NN200723 and evaluated its immunogenicity in comparison with commercial OEIVs derived from H120, 4/91, and QXL87 strains. All immunized groups exhibited significantly elevated specific and neutralizing antibody titers compared to non-immunized controls, with the GX-NN200723-OEIV group showing the highest responses. This enhanced immunogenicity is attributable to the molecular characteristics of GX-NN200723 characterized earlier: its S1 gene shares only 77.4% nucleotide identity with H120, and B-cell epitope prediction revealed distinct differences in antigenic sites between this strain and commonly used vaccine strains (H120, 4/91, QXL87, LDT3-A) [18]. These molecular differences result in limited antigenic relatedness between GX-NN200723 and commercial vaccine strains, explaining the poor cross-protection observed in commercial vaccine groups. In contrast, the homologous GX-NN200723-OEIV perfectly matches the challenge strain’s S1 gene and B-cell epitope profiles, enabling more specific and potent immune responses. Cell-dependent immunity analysis revealed significantly elevated CD3^+^, CD4^+^, and CD8^+^ T lymphocyte levels in all immunized groups versus non-immunized controls. At 28 dpv, CD3^+^ T cell increases exceeded those of CD4^+^ T cells, while CD8^+^ T cell activation was weakest – consistent with the known limitation of inactivated vaccines, which activate CD4^+^ T cells via exogenous antigen presentation but fail to engage endogenous pathways required for optimal CD8^+^ T cell activation [41,42]. This limitation, also reflected in Figure 12C, underscores an inherent trade-off of OEIV compared to live attenuated vaccines. IL-4 and IFN-γ levels (markers of Th2 and Th1 responses, respectively) were significantly elevated in all immunized groups, with GX-NN200723-OEIV showing levels comparable to commercial vaccine strains [43]. In conclusion, GX-NN200723-OEIV induced robust humoral and cell-dependent immunity, demonstrating strong immunogenicity as an inactivated vaccine. While OEIV offers clear safety advantages over live attenuated vaccines (avoiding risks of mutation and recombination), its suboptimal CD8+ T cell response highlights the need for future adjuvant optimization to enhance cell-dependent immunity.

In our study, all experimental groups except the control were challenged with the Taiwan-I-type IBV strain GX-NN200723 14 d post-booster immunization and monitored until 98 dpc (140 d of age), and chickens immunized with GX-NN200723-OEIV exhibited the most effective homologous protection, characterized by the mildest clinical signs and a 100% survival rate – this performance was comparable to a previously reported OEIV [44] and significantly superior to both the unvaccinated-challenged group and other commercial vaccine groups; gross lesion examination at 5 and 98 dpc further supported this protective efficacy, as the GX-NN200723-OEIV group, similar to the control group, had almost no tracheal or renal lesions, whereas unvaccinated-challenged chickens displayed severe tracheal hemorrhage, renal swelling, testicular atrophy, and reproductive tract impairment, and although the GX-NN200723-OEIV group showed slightly delayed reproductive development compared to the control group, this was still better than that in other immunized groups, with histopathological analysis confirming this advantage by showing no tracheal ciliary damage or renal inflammation in the GX-NN200723-OEIV group, which outperformed other vaccinated groups. Notably, no significant reproductive damage was detected in any experimental group at 98 dpc, and when combined with our pathogenicity data for GX-NN200723, this finding indicates that the strain’s reproductive tract damage occurs primarily within the first 14 dpi but still causes persistent developmental impairment in adult chickens, while inflammatory infiltration was absent by 98 dpc likely due to the host’s self-repair capacity. Consistent with these findings, viral load analysis confirmed GX-NN200723-OEIV’s strong homologous protection, as it resulted in the lowest viral loads in tracheal and renal tissues, reduced viral shedding, and a shorter duration of viral excretion – achieving an efficacy level comparable to that of current live attenuated vaccines [15–17]. To further optimize this vaccine, we will address OEIV’s limited induction of CD8^+^ T cell responses (corroborated by Figure 12C) by incorporating adjuvants designed to enhance cell-dependent immunity, which will synergistically strengthen CD8^+^ T cell responses critical for viral clearance while preserving the humoral immunity induced by OEIV, thereby refining IB control strategies. However, protective efficacy was only evaluated against homologous challenge in the present study, and assessment of heterologous protection represents a critical avenue for future investigation. Owing to the significant divergence in the S1 gene and distinct B-cell epitope profiles between GX-NN200723 and commonly used vaccine strains, cross-protection against heterologous IBV strains is expected to be limited, and thus the heterologous protective efficacy of GX-NN200723-OEIV remains a key direction for future investigation.

## Conclusions

In conclusion, our study provides a comprehensive characterization of the emerging Taiwan-I-type IBV strain GX-NN200723. Whole-genome analysis revealed its classical structure, characterized by a recombination origin and unique B-cell epitope profiles that explain the limited cross-protection offered by conventional vaccines. Pathogenicity assessment confirmed its broad tissue tropism, notably establishing for the first time its capacity to cause significant lesions in the male reproductive system (testicular atrophy) and to induce developmental delays in the female reproductive tract. Crucially, the Taiwan-I-type-specific OEIV developed in this study demonstrated robust immunogenicity, eliciting potent humoral and cell-dependent immune responses, and conferred superior protection against homologous challenge with GX-NN200723, outperforming several commercial vaccines. Notably, vaccine efficacy was only evaluated against homologous challenge in this study. These findings not only enhance our understanding of the molecular and pathogenic features of current Taiwan-I-type strains but also validate a promising, safe, and effective candidate vaccine, offering a practical and powerful tool for improving IB control strategies.

## Data Availability

All the data required to draw the conclusions in the paper have been presented in the main text. The original data of all the charts and tables have been stored in the Figshare database (https://doi.org/10.6084/m9.figshare.31242154 [45]).
